# Multisource Early Warning Framework Integrating Regional Pathogen Surveillance and Baidu Index Data for Respiratory Infectious Disease Prediction in Beijing: Retrospective Time-Series Modeling Study

**DOI:** 10.2196/100492

**Published:** 2026-09-11

**Authors:** Yang Liu, Yanlin Gao, Jing Du, Yunping Shi, Xiao Hu, Gang Li

**Affiliations:** 1 Division of Disease Burden Research, Big Data Center, Beijing Center for Disease Prevention and Control, 16 Hepingli Zhongjie, Dongcheng District, Beijing, 100013, China, 86 13521529956; 2 School of Public Health, Capital Medical University, Beijing, China

**Keywords:** respiratory infectious diseases, early warning, pathogen surveillance, Baidu index, multisource data fusion, LASSO-Poisson, least absolute shrinkage and selection operator

## Abstract

**Background:**

Respiratory infectious disease surveillance increasingly requires timely integration of routine case reporting, laboratory pathogen surveillance, and digital behavioral signals. However, the incremental predictive value of spatially stratified regional pathogen activity and internet search data for short-term urban respiratory disease prediction remains unclear.

**Objective:**

This study aimed to develop and initially validate an exploratory multisource prediction framework for short-term prediction and early warning of respiratory infectious disease activity in Beijing by integrating reported case data, spatially stratified regional pathogen surveillance indicators, and Baidu index search data.

**Methods:**

We conducted a retrospective time-series modeling study using weekly reported respiratory infectious disease cases in Beijing, regional pathogen surveillance data from 31 provincial-level administrative regions in China, and Baidu index search data from January 2023 to December 2025. Pathogen indicators were aggregated by spatial strata and lagged by 0 to 4 weeks. Ten candidate LASSO (least absolute shrinkage and selection operator)–Poisson models were constructed to compare historical case terms, seasonal terms, local and regional pathogen indicators, and Baidu index indicators. One-week-ahead prediction was the primary task. Models were evaluated using a chronological train-test split and rolling-origin cross-validation for penalty parameter selection. Performance was assessed using root mean squared error (RMSE), mean absolute error (MAE), symmetric mean absolute percentage error (SMAPE), and RMSE reduction relative to the baseline historical case model.

**Results:**

The M10 core multisource model achieved the lowest overall RMSE among all candidate models, with an RMSE of 16,658.24; an MAE of 10,303.07; and an SMAPE of 0.766, corresponding to a 73.5% RMSE reduction relative to the M1 baseline model. The M3 regional pathogen model ranked second by RMSE, whereas the M7 Baidu index model achieved the lowest SMAPE, indicating better relative performance during low-burden periods. Selected predictors included regional overall pathogen positivity, influenza A positivity, coinfection-related indicators, historical case terms, and Baidu index disease name search indicators.

**Conclusions:**

The core multisource model improved the 1-week-ahead prediction of respiratory infectious disease case counts in Beijing, although relative prediction performance differed across models. Spatially stratified regional pathogen activity indicators, particularly overall pathogen positivity, influenza A positivity, and coinfection-related indicators, served as important supplementary signals, while Baidu index data provided complementary digital information. These findings support the feasibility of an exploratory multisource prediction framework in Beijing. Further prospective temporal validation and spatial validation in other megacities are needed before broader application. The core multisource model improved 1-week-ahead absolute case count prediction of respiratory infectious diseases in Beijing according to RMSE but did not achieve the lowest relative error according to SMAPE. These findings support the feasibility of an exploratory multisource prediction framework in Beijing. Further prospective temporal validation and spatial validation in other megacities or urban settings are needed before broader application.

## Introduction

Respiratory infectious diseases are characterized by rapid transmission, marked seasonal fluctuations, and the potential to cause sudden increases in health care service demand within a short period. They have therefore remained a major focus of public health surveillance and emergency response. For megacities with high population density, frequent population mobility, and complex social contact networks, the risks of transmission and the pressure on prevention and control are even more pronounced. Timely identification of abnormal increases in disease activity is therefore essential for public health emergency preparedness, health care resource allocation, and early intervention. Traditional surveillance based on reported cases can directly reflect disease occurrence, but delays in health care seeking, diagnosis, reporting, and data aggregation often limit its ability to capture early signals before an epidemic upswing. In recent years, early warning for infectious diseases has gradually shifted from single-source case surveillance toward multisource data integration. Laboratory-based pathogen surveillance, syndromic surveillance, internet search behavior, and other digital signals have been used to improve the sensitivity and timeliness of surveillance [1-3]. Internationally, laboratory surveillance networks such as the World Health Organization Global Influenza Surveillance and Response System (GISRS) and the United States National Respiratory and Enteric Virus Surveillance System (NREVSS) continuously collect pathogen testing data to track respiratory pathogen activity, highlighting the fundamental role of pathogen surveillance in respiratory infection surveillance systems [4,5].

Pathogen surveillance can provide an important supplement to case reporting surveillance. Compared with reported cases, pathogen positivity rates, overall positivity rates, and coinfection-related indicators may more directly reflect the circulation intensity of respiratory pathogens in the population. Previous studies have used influenza virus positivity rates, influenza-like illness proportions, and other laboratory surveillance indicators to predict influenza activity, suggesting that pathogen-based indicators can provide useful information for assessing short-term epidemic trends [6,7]. In addition, the transmission of respiratory pathogens exhibits clear spatiotemporal dependence. Interregional population mobility, geographic proximity, and similar climatic and seasonal conditions may enable pathogen activity in one region to provide leading signals for changes in cases in another region. Previous research has shown that incorporating spatial transmission information into prediction frameworks can improve the forecasting of regional spread of influenza and other respiratory diseases [8]. However, existing studies have often used pathogen data as either local indicators or broad national aggregate indicators, and few have systematically compared the relative contributions of pathogen signals at different spatial scales, such as local areas, neighboring provinces, and regions with similar climatic characteristics, to city-level respiratory infectious disease early warning. Regional pathogen indicators constructed through spatial stratification may be more useful for identifying upstream signals associated with epidemic changes in the target city.

Meanwhile, digital surveillance data, such as internet search behavior, have also been widely used for infectious disease surveillance and prediction. Previous studies have demonstrated that Baidu search data can be used to monitor influenza activity in China. Subsequent research has further combined Baidu search data with traditional influenza-like illness or virological surveillance data to predict influenza epidemic trends and support early detection [6]. Similarly, internet search data such as Google Trends have been used for digital epidemiological surveillance of influenza, COVID-19, and other infectious diseases [9,10]. However, internet search data also have notable limitations. They may be affected by media coverage, changes in public attention, search platform algorithm adjustments, and non–disease-related factors. Reliance on search behavior alone may therefore produce unstable or even misleading predictions [1,10,11]. Accordingly, search data are better suited as a complement to traditional case reporting and laboratory-based pathogen surveillance rather than as a replacement. Compared with individual keywords, composite search indicators constructed based on clinical relevance can help reduce keyword-specific noise, improve interpretability, and decrease instability caused by multiple comparisons.

Although pathogen surveillance and internet search data have both shown potential for infectious disease prediction, several gaps remain in the existing literature. First, many studies have focused on a single disease or pathogen, such as influenza or COVID-19, whereas fewer have addressed broader surveillance outcomes covering notifiable respiratory infectious diseases. Second, previous models have mostly used local cases, local pathogen indicators, or national aggregate data, and systematic comparisons of regional pathogen activity signals at different spatial scales remain limited. Third, although search behavior data are timely, they are susceptible to external events and may be unstable when used alone. Joint modeling studies that integrate spatially stratified regional pathogen surveillance, search behavior data, and historical case trends within a unified framework remain relatively limited. Fourth, for megacities such as Beijing, which are characterized by high population density, substantial mobility, concentrated health care resources, and considerable pressure for respiratory infectious disease prevention and control, there is still insufficient empirical evidence on how to use pathogen surveillance information from 31 provincial-level administrative regions in China to construct spatially stratified indicators and integrate them with local Baidu index data for city-level early warning.

To address these gaps, this study developed a multisource fusion early warning framework for respiratory infectious diseases in Beijing by integrating reported case data, national provincial-level pathogen surveillance data, and Baidu index search data. We constructed regional pathogen activity indicators at multiple spatial scales and grouped Baidu search terms into clinically meaningful composite indicators. We then evaluated lagged correlations between multisource indicators and reported cases in Beijing and compared the short-term predictive performance of historical case baseline models, regional pathogen models, Baidu index models, and multisource fusion models. This study aimed to assess the incremental value of spatially stratified regional pathogen activity and Baidu search behavior for short-term prediction of respiratory infectious disease activity in Beijing, providing empirical evidence for multisource early warning frameworks in megacities.

## Methods

### Study Design and Data Sources

This study used a retrospective weekly time series design to evaluate the value of multisource surveillance indicators for short-term prediction and early warning of respiratory infectious disease activity in Beijing. Three categories of data were integrated: weekly reported notifiable respiratory infectious disease cases in Beijing from January 2023 to December 2025, weekly pathogen surveillance data from 31 provincial-level regions in China during the same period, and daily Baidu index search data for Beijing from January 2023 to December 2025.

The primary outcome was the weekly number of reported notifiable respiratory infectious disease cases in Beijing. Case data were aggregated by epidemiological week, and the week start date was used as the temporal index for data integration. Province-level pathogen surveillance data included weekly specimen testing volume, pathogen-specific detections, single-pathogen detections, and coinfection-related detections. Daily Baidu index data were aggregated to the weekly level and aligned with the same week start date.

### Construction of Spatially Stratified Regional Pathogen Indicators

To evaluate the predictive value of pathogen activity at different spatial scales for changes in respiratory infectious diseases in Beijing, we constructed spatially stratified regional pathogen indicators based on weekly pathogen surveillance data from 31 provincial-level administrative regions in China. The regional groupings included local pathogen indicators for Beijing, neighboring-province indicators, northern-China indicators excluding Beijing, southern-China indicators, and national indicators excluding Beijing (see Multimedia Appendix 1).

For each region and each surveillance week, we aggregated the number of tests, pathogen detections, single-pathogen detections, and coinfection-related detections across all provinces within the region and calculated regional-level pathogen activity indicators. The regional pathogen positivity rate was calculated as the total number of pathogen detections within the region divided by the total number of tests within the region, to reduce potential bias caused by differences in testing volume across provinces. The formula was as follows:


$$\begin{array}{r} \text{Regional pathogen positivity rate (\textbackslash{}\%)}\\ =\left(\frac{\text{total number of pathogen detections within the region}}{\text{total number of tests within the region}}\right)\\ \;\times 100 \end{array}$$

The same aggregation principle was applied to pathogen-specific positivity rates, single-pathogen positivity rates, coinfection-related positivity rates, and coinfection rates. The main indicators included pathogen-specific positivity rates, overall pathogen positivity rate, single-pathogen positivity rate, coinfection-related positivity rate, and coinfection rate. Pathogen-specific positivity rates mainly included the positivity rates of influenza A virus, influenza B virus, and SARS-CoV-2. The overall pathogen positivity rate was used to reflect the general level of respiratory pathogen activity in the region, whereas coinfection-related indicators were used to reflect the complexity of concurrent circulation of multiple pathogens. All regional pathogen indicators were matched with weekly reported cases of notifiable respiratory infectious diseases in Beijing according to the week start date. Lagged variables from 0 to 4 weeks were further generated for lagged correlation analysis and predictive model development.

### Construction of Baidu Index Composite Indicators

Baidu index search terms were grouped according to clinical relevance and epidemiological plausibility. The core respiratory symptom group included cough, fever, dyspnea, shortness of breath, sore throat, and throat pain. The respiratory disease name group included influenza, respiratory tract infection, COVID-19, and recurrent positivity. The nonspecific accompanying symptoms group included nausea, hemoptysis, chest pain, and headache. The detailed grouping of Baidu index search terms is shown in Multimedia Appendix 2.

The first 2 groups were used as core Baidu index indicators in the main prediction models, whereas the nonspecific accompanying symptoms group was mainly used for supplementary analyses. For each keyword, daily Baidu index values were aggregated to weekly values. To reduce the influence of different search volume scales across keywords, keyword-specific weekly series were standardized before constructing group-level composite indicators. The composite index for each group was calculated as the mean of standardized weekly values across terms in that group.

### Lagged Correlation Analysis

Lagged correlation analysis was conducted to assess temporal associations between multisource surveillance indicators and weekly reported respiratory infectious disease cases in Beijing. The spatial grouping of regional pathogen surveillance indicators and the grouping of Baidu index search terms are provided in Multimedia Appendix 1 and Multimedia Appendix 2, respectively. Spearman correlation coefficients were calculated between each surveillance indicator and weekly Beijing case counts at lags of 0 to 4 weeks. Lag 0 represented same-week correlation, whereas lags 1 to 4 indicated that the surveillance indicator preceded Beijing case counts by 1 to 4 weeks, respectively. Heat maps were used to visualize the direction and magnitude of correlations across lag weeks. For each indicator, the best lag was defined as the lag within the range of 0 to 4 weeks with the largest absolute unrounded Spearman correlation coefficient. Best-lag correlation plots were then used to summarize indicators showing strong synchronous or potentially leading associations. The findings from these exploratory analyses were used to inform the selection of indicators and lag structures for the core multisource prediction model.

### Prediction Model Development

In this study, to evaluate the incremental value of different surveillance data sources and their spatially stratified indicators for the short-term prediction of respiratory infectious diseases in Beijing, we used LASSO (least absolute shrinkage and selection operator)–Poisson regression with a log link as a unified prediction framework. This method was selected for the following reasons. First, the study outcome was weekly reported case counts, which are count data; therefore, the Poisson model provides good interpretability. Second, the candidate predictors included 0- to 4-week lagged variables from multiple data sources, resulting in a relatively large number of variables with potential multicollinearity. LASSO regularization can perform variable shrinkage and selection, thereby reducing the risk of overfitting. Third, the primary objective of this study was to compare the incremental predictive value of different surveillance data sources and their spatial structures for short-term prediction rather than to compare the performance of different algorithms. Using a unified modeling framework therefore helps ensure comparability across candidate models. Based on this framework, we developed 10 candidate LASSO-Poisson prediction models. The baseline model included historical case features in Beijing, namely case counts lagged by 1 to 4 weeks, a 4-week moving average, and seasonal sine and cosine terms. Other models additionally incorporated Beijing local pathogen indicators, national pathogen indicators excluding Beijing, spatially stratified regional pathogen indicators, Baidu index composite indicators, and multisource combined indicators on top of the baseline model. The definitions of all candidate models and their input data sources are summarized in Table 1.

**Table 1.** Definition of candidate prediction models and input data sources.

Model	Model type	Input data
M1 baseline	Historical case model	Lagged Beijing case counts, 4-week moving average, and seasonal terms
M2 Beijing pathogen	Local pathogen model	M1 + Beijing pathogen indicators
M3 national excluding Beijing	National background pathogen model	M1 + national pathogen indicators excluding Beijing
M4 northern region	Regional pathogen model	M1 + northern-region pathogen indicators
M5 neighboring provinces	Regional pathogen model	M1 + neighboring-province pathogen indicators
M6 southern control	Distant-region control model	M1 + southern-region pathogen indicators
M7 Baidu index	Digital surveillance model	M1 + Baidu index composite indicators
M8 regional + Baidu	Regional-digital model	M1 + northern-region, neighboring-province, and national excluding Beijing pathogen indicators + Baidu index indicators
M9 full multisource	Full multisource model	M1 + all candidate pathogen and Baidu index indicators
M10 core multisource	Core multisource model	M1 + selected regional pathogen indicators + core Baidu index indicators

For each external surveillance indicator, lagged predictors from 0 to 4 weeks were generated. For 1-week-ahead prediction, predictors observed up to week t were used to predict reported case counts in week *t*+1. Lag 0 represented information from the prediction-origin week t, whereas lags 1 to 4 represented information from weeks t−1 to t−4, respectively. No backfilling was used for lagged predictors. Because the candidate models included 1- to 4-week lagged case count features and 0- to 4-week lagged external surveillance indicators, observations without sufficient lagged information at the beginning of the study period were excluded through complete-case filtering before the chronological train-test split. In practice, this resulted in the exclusion of the first 4 calendar weeks. This filtering step was applied to the unified modeling dataset before constructing the final candidate models; thus, all models were fitted and evaluated using the same effective study period and the same chronological train-test split. Weeks without sufficient lagged information were excluded consistently before model fitting. LASSO regularization was used to select informative predictors from the high-dimensional lagged feature set and to reduce model overfitting.

The data were split chronologically, with the first 80% of weeks used for training and the remaining 20% used for out-of-sample testing. This chronological split was used to preserve the time series structure and avoid information leakage caused by random splitting. Within the training set, the penalty parameter was selected using rolling-origin cross-validation. At each validation step, the model was fitted using only earlier weeks and evaluated on subsequent validation weeks, thereby preserving temporal ordering and avoiding information leakage that could occur with random k-fold cross-validation in autocorrelated time series data. The lambda value with the lowest mean validation error was selected as lambda.min, and the final model was refitted on the full training set using the selected lambda. Details of the selected lambda values, lambda selection rule, number of rolling validation splits, and number of nonzero predictors are provided in Multimedia Appendix 3.

### Model Performance Evaluation

Model performance for 1-week-ahead case count prediction was evaluated in the held-out test set. Root mean squared error (RMSE) was used as the primary ranking metric because the main prediction target was weekly case count burden. Mean absolute error (MAE) and symmetric mean absolute percentage error (SMAPE) were reported as complementary error metrics. RMSE reduction relative to the baseline model was calculated to quantify the incremental value of additional surveillance data sources. Positive values indicated lower RMSE than the baseline model, whereas negative values indicated higher RMSE. To further examine whether differences between RMSE-based and SMAPE-based rankings were related to epidemic burden level, we conducted an additional burden-stratified performance analysis in the held-out test set. Test weeks were classified as high-burden weeks if their observed weekly case counts were greater than or equal to the 75th percentile of observed case counts in the held-out test set. The remaining test weeks were classified as low-burden weeks. RMSE, MAE, and SMAPE were then recalculated separately within each burden stratum. This analysis was used to explore the performance of candidate models during high-activity and low-activity periods and was not used for model selection.

### Sensitivity Analysis Across Forecast Horizons

To assess whether the predictive value of multisource surveillance indicators persisted over a longer forecast horizon, we conducted a 2-week-ahead prediction sensitivity analysis. The same modeling framework, candidate model definitions, and chronological train-test split used in the primary 1-week-ahead analysis were applied, but the outcome was changed from 1-week-ahead case counts to 2-week-ahead case counts. Model performance was evaluated using RMSE, MAE, SMAPE, and RMSE reduction relative to the M1 baseline model and was compared with the primary 1-week-ahead prediction results. This analysis was used to assess the robustness of model performance when the forecast horizon was extended.

### Ethical Considerations

This study used aggregated routine public health surveillance data, regional pathogen surveillance data, and Baidu index data. No individual-level identifiable information was included in the analysis, and no participants were recruited or assigned to any intervention. According to relevant institutional and local public health data governance requirements, analyses based exclusively on aggregated routine surveillance data without individual identifiers do not require additional ethics committee review. The requirement for informed consent was therefore waived. All data were analyzed in aggregated form, and protective measures were used to safeguard confidentiality during data management and analysis. No participant compensation was provided because no participants were recruited for this study.

## Results

### Temporal Patterns of Multisource Surveillance Indicators

#### Temporal Trends in Weekly Reported Cases of Notifiable Respiratory Infectious Diseases in Beijing

**Figure 1.**
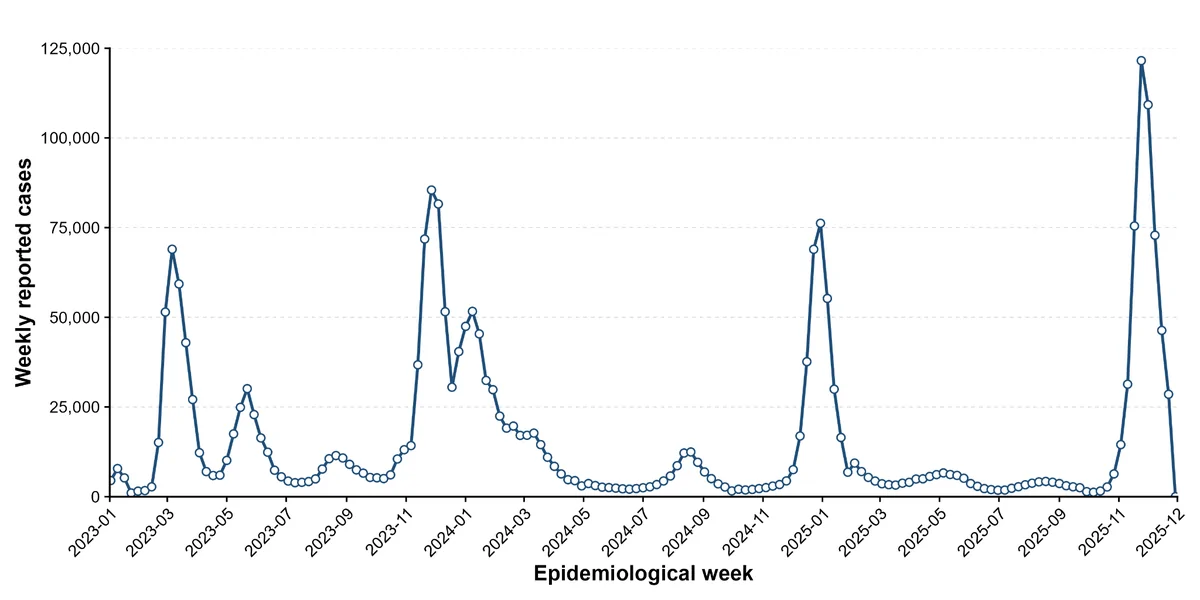
Weekly reported cases of notifiable respiratory infectious diseases in Beijing from January 2023 to December 2025.

From January 2023 to December 2025, weekly reported cases of notifiable respiratory infectious diseases in Beijing showed clear seasonal fluctuations. Multiple episodic peaks were observed during the study period, with an overall relatively stable bimodal seasonal pattern characterized by a major winter peak and a secondary spring peak. The major peak mainly occurred from November to January of the following year, whereas the secondary peak generally appeared around March. In contrast, case counts remained relatively low during summer and autumn. The most prominent winter peak during the observation period occurred from November to December 2025 (Figure 1).

#### Temporal Patterns of Overall Pathogen Positivity Rates Across Regions

**Figure 2.**
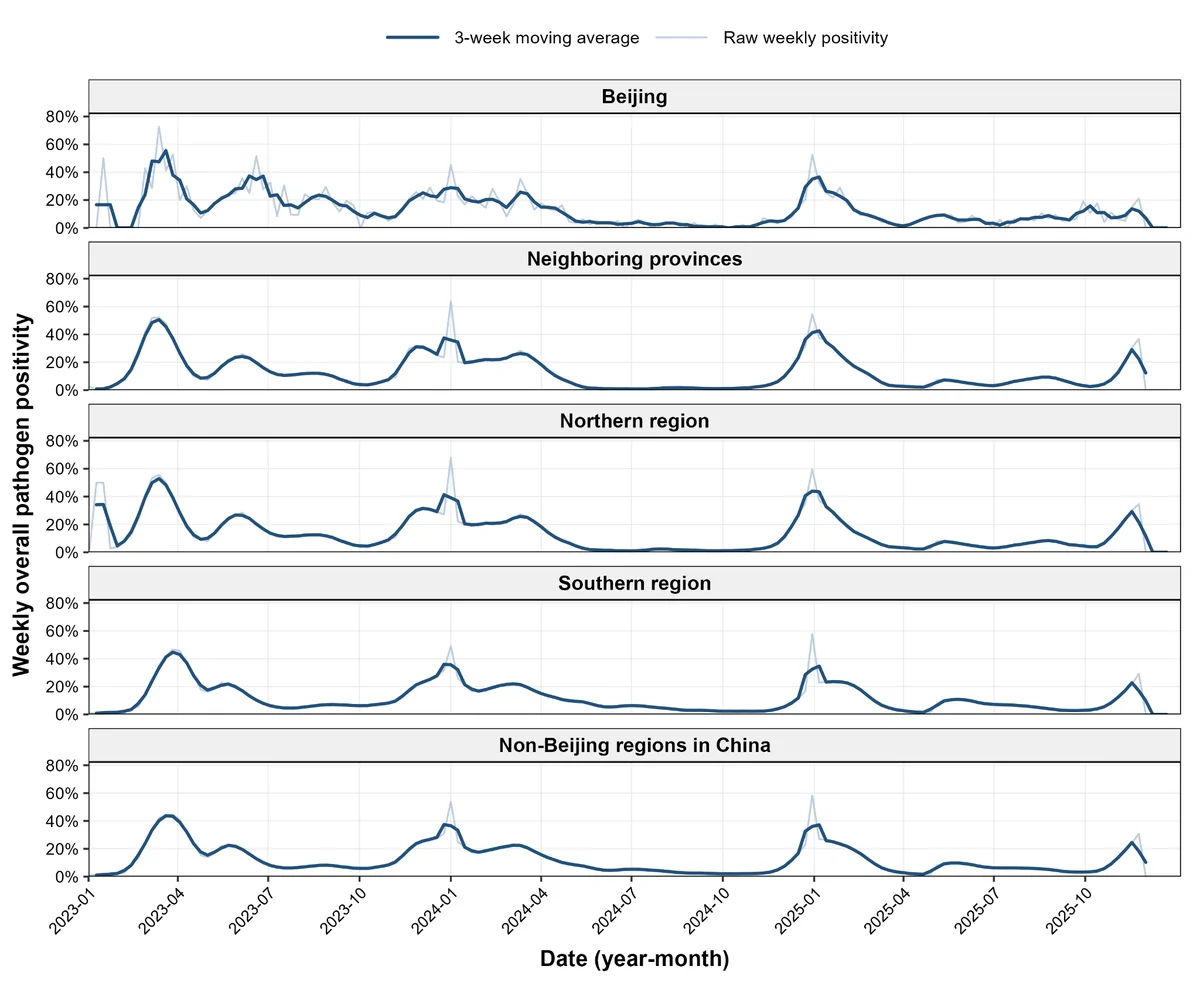
Weekly overall pathogen positivity across regions with 3-week moving average trends, 2023‐2025. Light blue lines represent raw weekly positivity rates, and dark blue lines represent 3-week moving averages. Positivity rates were calculated as the total number of pathogen detections divided by the total number of specimens tested within each region-week.

From January 2023 to December 2025, weekly overall pathogen positivity rates showed clear temporal fluctuations across regions. Overall pathogen positivity rates in Beijing, neighboring provinces, northern region, southern region, and national data excluding Beijing were generally higher during winter and spring and lower during summer and autumn. Although the overall temporal patterns were broadly similar across regions, differences were observed in peak intensity and timing. The Beijing local indicator showed more frequent short-term fluctuations, whereas indicators from neighboring provinces, northern region, and national data excluding Beijing appeared smoother, reflecting broader regional pathogen activity. These findings indicate that regional pathogen surveillance indicators captured seasonal variation in respiratory pathogen circulation and provided a basis for subsequent lagged correlation analysis and prediction modeling (Figure 2).

#### Temporal Trends in Baidu Index Composite Indicators

**Figure 3.**
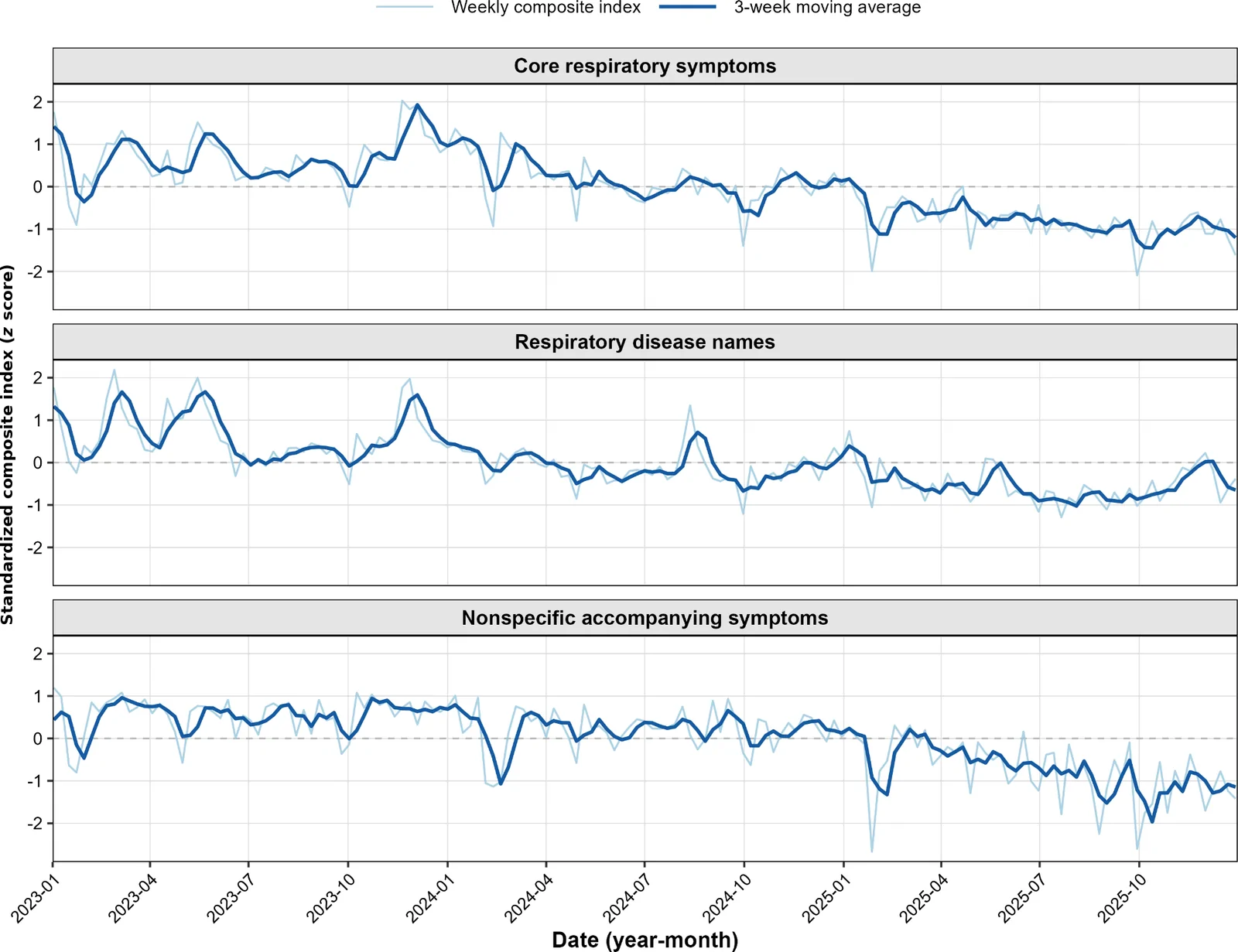
Weekly trends of Baidu index composite indicators in Beijing. Light blue lines represent the raw weekly composite indices, and dark blue lines represent the 3-week moving averages. Search terms were grouped into core respiratory symptoms, respiratory disease names, and nonspecific accompanying symptoms according to clinical relevance.

The Baidu index composite indicators in Beijing showed clear temporal variation from January 2023 to December 2025 (Figure 3). The core respiratory symptom group and respiratory disease name group were at relatively high levels at the beginning of 2023, declined during January to February, and increased again around March 2023 and in late 2023, followed by a decline in early 2024. During 2024, both indicators showed intermittent short-term fluctuations; the respiratory disease name group also showed a transient increase around September 2024. From 2025 onward, the scores in the 2 respiratory-related groups generally remained below their earlier peak levels and showed an overall downward pattern toward the end of the study period, although a small rebound in the respiratory disease name group was observed around late 2025. The nonspecific accompanying symptoms group showed weaker fluctuations and less distinct peak patterns in the composite scores than the 2 respiratory-related groups, although the nonspecific accompanying symptoms group scores also declined after early 2025. These findings indicate that respiratory symptom and disease name search composites displayed more distinct temporal patterns than nonspecific accompanying symptoms.

### Lagged Correlations Between Regional Pathogen Activity and Beijing Cases

**Figure 4.**
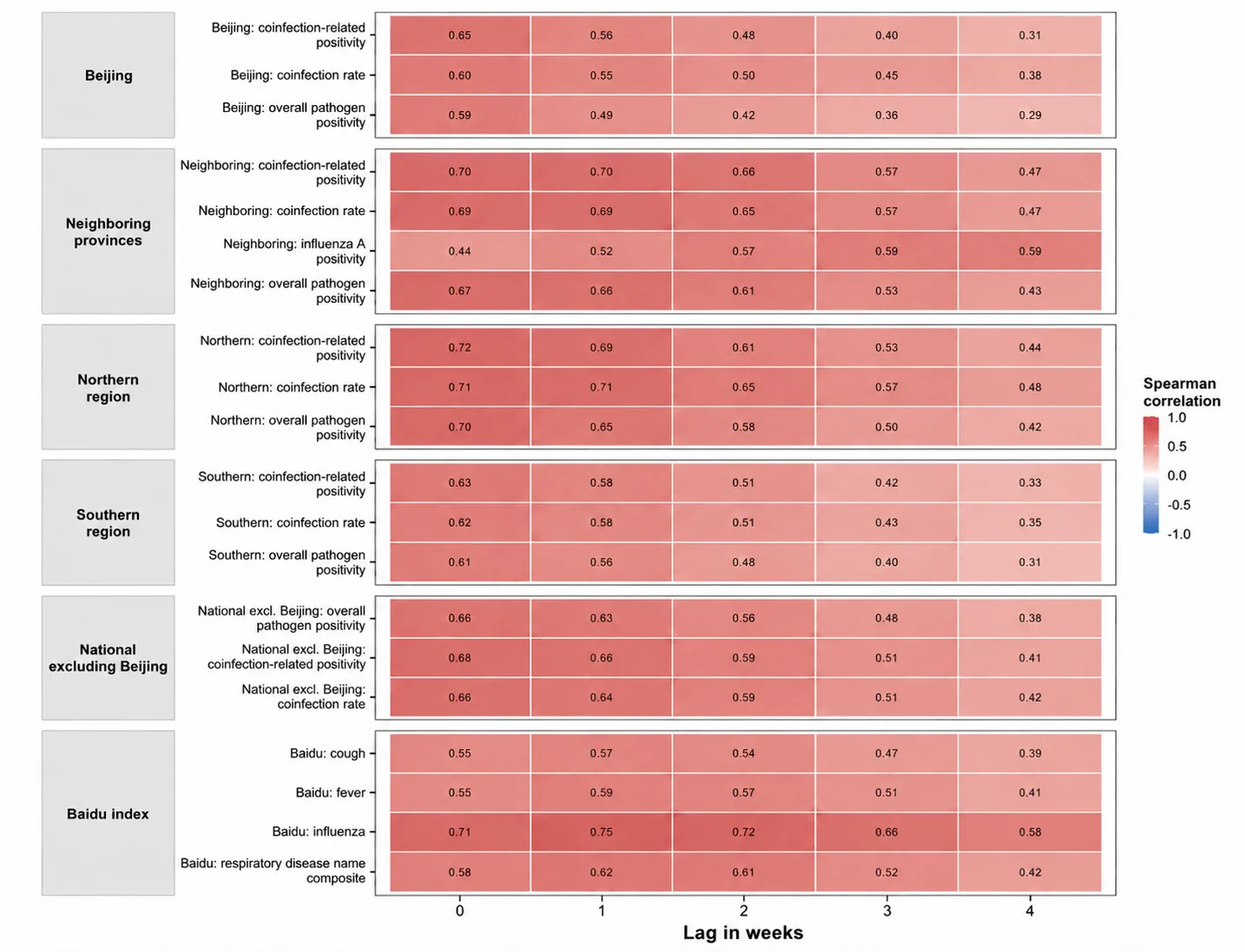
Lagged correlations between regional pathogen activity indicators and weekly reported notifiable respiratory infectious disease cases in Beijing. Spearman correlation coefficients were calculated between each surveillance indicator and weekly case counts at lags of 0‐4 weeks. Lag 0 indicates same-week correlation, whereas lags 1‐4 indicate that the surveillance indicator preceded Beijing case counts by 1‐4 weeks. Red indicates positive correlations and blue indicates negative correlations. Only core respiratory-related indicators are shown. excl.: excluding.

Lagged correlation analysis showed that the selected regional pathogen activity indicators were positively correlated with weekly reported respiratory infectious disease cases in Beijing across lags of 0 to 4 weeks (Figure 4). At lag 0, the strongest correlations were observed in the northern region, where coinfection-related positivity, coinfection rate, and overall pathogen positivity had Spearman correlation coefficients of 0.72, 0.71, and 0.70, respectively. The corresponding coefficients were 0.70, 0.69, and 0.67 in neighboring provinces and 0.68, 0.66, and 0.66 in national non-Beijing regions. For most overall pathogen activity and coinfection-related indicators, correlations decreased as the lag increased. Influenza A positivity in neighboring provinces showed a different pattern, with correlation coefficients increasing from 0.44 at lag 0 to 0.52, 0.57, 0.59, and 0.59 at lags 1 to 4, respectively. Baidu index indicators also showed leading correlations. The Baidu influenza search index reached its highest correlation at lag 1 (ρ=0.75), while the cough, fever, and respiratory disease name composite indices also peaked at lag 1, with coefficients of 0.57, 0.59, and 0.62, respectively.

**Figure 5.**
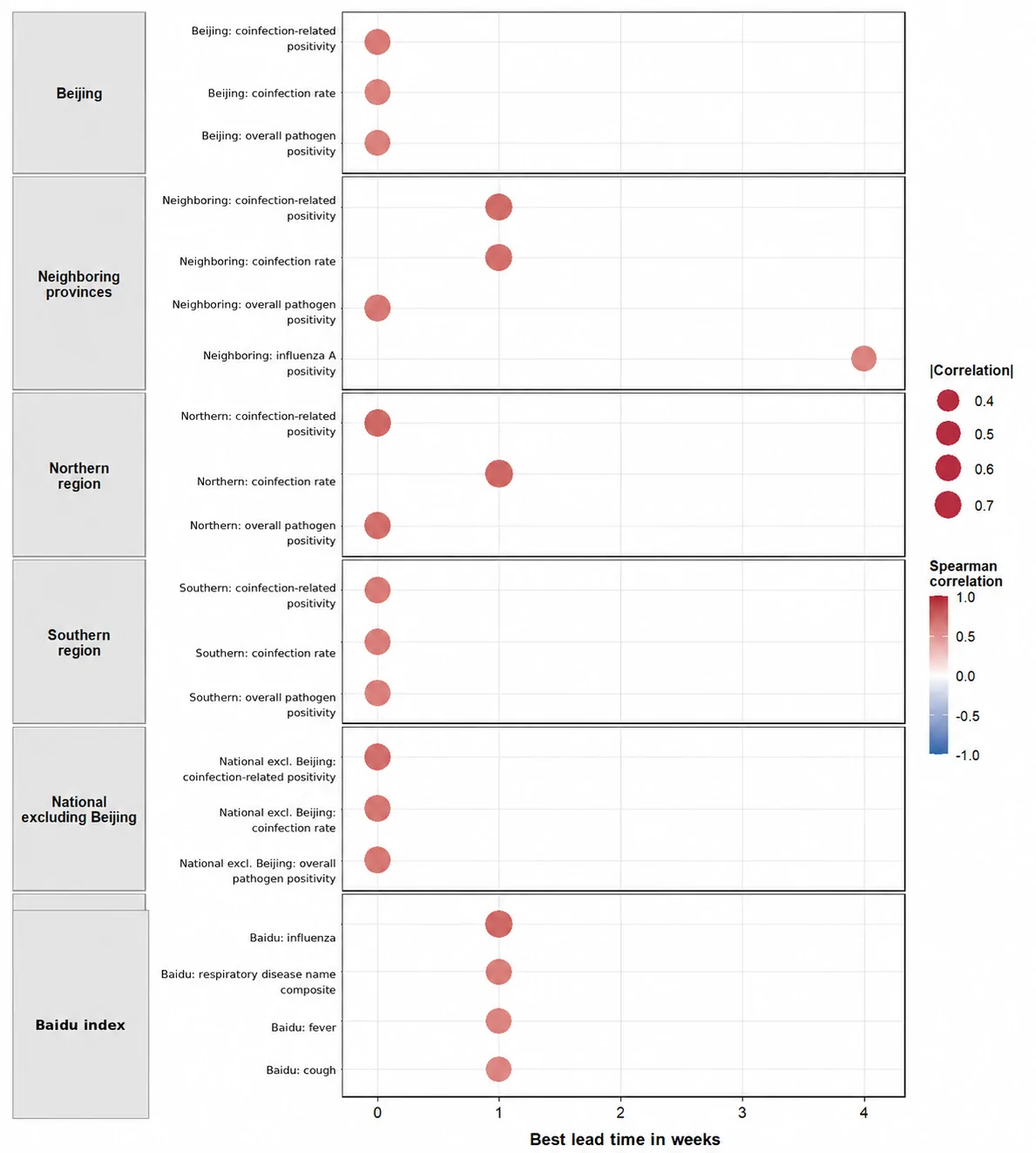
Optimal lead time of multisource surveillance indicators based on lagged Spearman correlation analysis. For each surveillance indicator, Spearman correlation coefficients with weekly respiratory infectious disease case counts in Beijing were calculated over a 0- to‐4-week lead time range. The lead time with the largest absolute correlation coefficient was selected as the optimal lead time. The x-axis indicates the optimal lead time, with 0 weeks representing the same week. The y-axis shows different surveillance indicators. Point size represents the absolute correlation coefficient, and color represents the direction and magnitude of the Spearman correlation, with red indicating positive correlations and blue indicating negative correlations. excl.: excluding.

Figure 5 showed that the optimal lead time was 0 weeks for most regional pathogen indicators. Coinfection-related positivity and coinfection rate in neighboring provinces, coinfection rate in the northern region, and all 4 Baidu index indicators had an optimal lead time of 1 week. Neighboring province influenza A positivity had the longest optimal lead time, at 4 weeks.

### Prediction Performance of Candidate Models

All candidate models were fitted using the same chronological train-test split and rolling-origin cross-validation procedure for penalty parameter selection. Based on the candidate model definitions in Table 1, we compared the 1-week-ahead predictive performance of the LASSO-Poisson models in the held-out test set. The 1-week-ahead prediction performance of all candidate models is summarized in Table 2. The M10 core multisource model achieved the lowest RMSE among all candidate models, with an RMSE of 16,658.24; an MAE of 10,303.07; and a SMAPE of 0.766, corresponding to a 73.5% RMSE reduction relative to the M1 baseline model. The M3 national excluding Beijing model also showed a large RMSE reduction of 71.7%, followed by the M6 southern control model, M5 neighboring province model, M8 regional pathogen plus Baidu index model, M9 full multisource model, and M7 Baidu index model.

**Table 2.** One-week-ahead prediction performance of candidate models.^a^Models were ranked by RMSE. RMSE reduction was calculated relative to the M1 baseline model. SMAPE was reported as a relative error metric and was not used as the primary ranking criterion.

Model	RMSE^b^	MAE^c^	SMAPE^d^	RMSE reduction^e^ (%)
M10 core multisource	16,658.24	10,303.07	0.766	73.5
M3 national excluding Beijing	17,843.03	13,831.38	1.005	71.7
M6 southern control	24,909.84	12,757.39	0.754	60.4
M5 neighboring provinces	26,914.69	18,356.43	1.076	57.3
M8 regional + Baidu	27,835.80	14,126.51	0.835	55.8
M9 full multisource	27,839.38	14,226.24	0.848	55.8
M7 Baidu index	27,976.11	10,926.24	0.512	55.6
M4 northern region	32,399.31	20,647.50	1.013	48.5
M2 Beijing pathogen	33,351.77	13,415.59	0.735	47.0
M1 baseline	62,964.98	18,788.72	0.714	0

a Models were ranked by root mean squared error (RMSE). SMAPE was reported as a relative error metric and was not used as the primary ranking criterion.

b RMSE: root mean squared error.

c MAE: mean absolute error.

d SMAPE: symmetric mean absolute percentage error.

e RMSE reduction was calculated relative to the M1 baseline model.

Although M10 achieved the lowest RMSE, it did not have the lowest SMAPE. The M7 Baidu index model had the lowest SMAPE, at 0.512, whereas the M10 model had a SMAPE of 0.766. These findings indicate that RMSE and SMAPE captured different aspects of model performance. Therefore, the models were ranked primarily by RMSE, while SMAPE was reported as a complementary relative error metric.

To further examine the divergence between RMSE and SMAPE, we conducted a burden-stratified performance analysis in the held-out test set (Table S1 Multimedia Appendix 4). Test weeks were divided into high-burden weeks and low-burden weeks according to the 75th percentile of observed weekly case counts. In high-burden weeks, the M10 core multisource model substantially reduced absolute prediction errors compared with the M1 baseline model, with RMSE decreasing from 126,000 to 31,500 and MAE decreasing from 63,635 to 27,238. However, M10 was not the best-performing model within this stratum; the M3 national excluding Beijing model had the lowest RMSE in high-burden weeks.

In low-burden weeks, M10 showed higher RMSE and SMAPE than the M1 baseline model. The RMSE and SMAPE were 6170 and 0.835, respectively, for M10, compared with 5640 and 0.762, respectively, for M1. By contrast, the M7 Baidu index model showed the lowest RMSE and SMAPE in low-burden weeks, at 3680 and 0.456, respectively. These findings suggest that the overall RMSE advantage of M10 was mainly driven by reductions in large absolute errors during high-burden weeks, whereas its relative prediction accuracy was less favorable during low-burden weeks.

### Model Interpretation: Selected Predictors in the M10 Core Multisource Model

**Figure 6.**
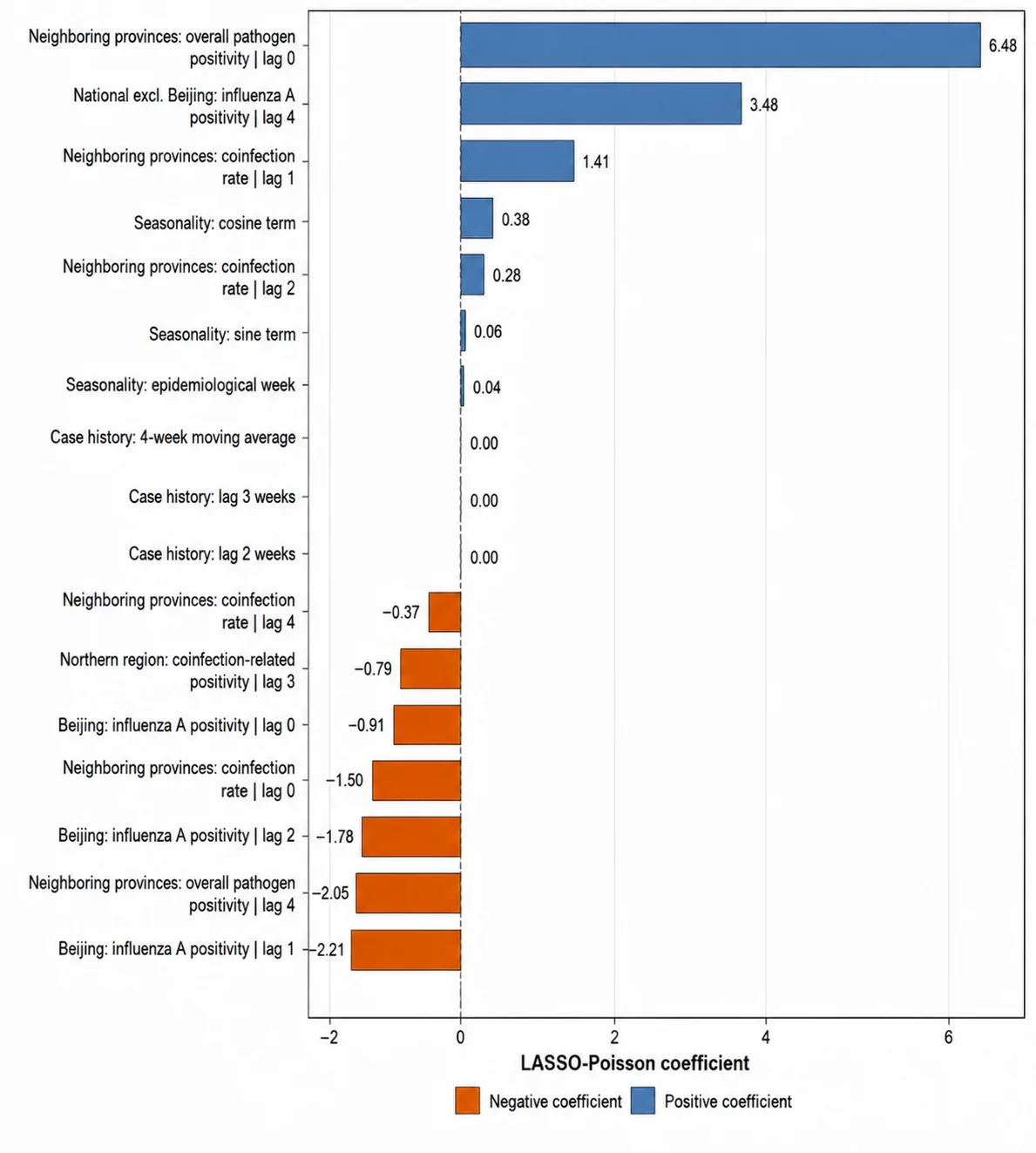
Selected predictors in the updated M10 core multisource LASSO-Poisson model. Bars show nonzero coefficients selected by the rolling-origin cross-validated LASSO-Poisson model. Positive coefficients indicate variables associated with higher predicted case counts after adjustment for other selected predictors, whereas negative coefficients indicate inverse adjustment directions within the penalized multivariable model. Coefficients should be interpreted as model-based predictive contributions rather than causal effects. LASSO: least absolute shrinkage and selection operator.

To interpret the variable composition of the M10 core multisource model, we extracted predictors with nonzero coefficients from the LASSO-Poisson model selected using rolling-origin cross-validation. The results showed that the variables retained in M10 were dominated by regional pathogen indicators, with a small number of historical case terms and seasonal terms also included (Figure 6).

Ranked by the absolute values of their coefficients, the leading positive predictors included same-week overall pathogen positivity in neighboring provinces, the 4-week lagged influenza A positivity in national non-Beijing regions, and the 1-week lagged coinfection rate in neighboring provinces. By contrast, although the seasonal cosine term, seasonal sine term, epidemiological week, and historical case terms were also retained, their coefficient magnitudes were relatively smaller.

Some selected predictors had negative coefficients, mainly including same-week and 1‐ to 2-week lagged Beijing influenza A positivity, same-week and 4-week lagged coinfection rate in neighboring provinces, 4-week lagged overall pathogen positivity in neighboring provinces, and 3-week lagged coinfection-related positivity in northern region. Overall, the selected variables in M10 were mainly concentrated in overall pathogen positivity, influenza A positivity, and coinfection-related indicators, suggesting that pathogen surveillance signals from different spatial sources and lag periods contributed differently to 1-week-ahead case count prediction.

### Sensitivity Analysis Across Forecast Horizons

To assess whether the predictive value of multisource surveillance indicators persisted over a longer forecast horizon, we conducted a 2-week-ahead prediction sensitivity analysis and compared the RMSE reductions across 1-week-ahead and 2-week-ahead forecasts.

For 1-week-ahead prediction, the M10 core multisource model achieved the largest RMSE reduction relative to the M1 baseline model, with a reduction of 73.5%. This was followed by the M5 neighboring province pathogen model, with an RMSE reduction of 57.3%. The M8 regional pathogen plus Baidu index model and the M9 full multisource model both reduced RMSE by 55.8%, while the M7 Baidu index model reduced RMSE by 55.6%.

**Figure 7.**
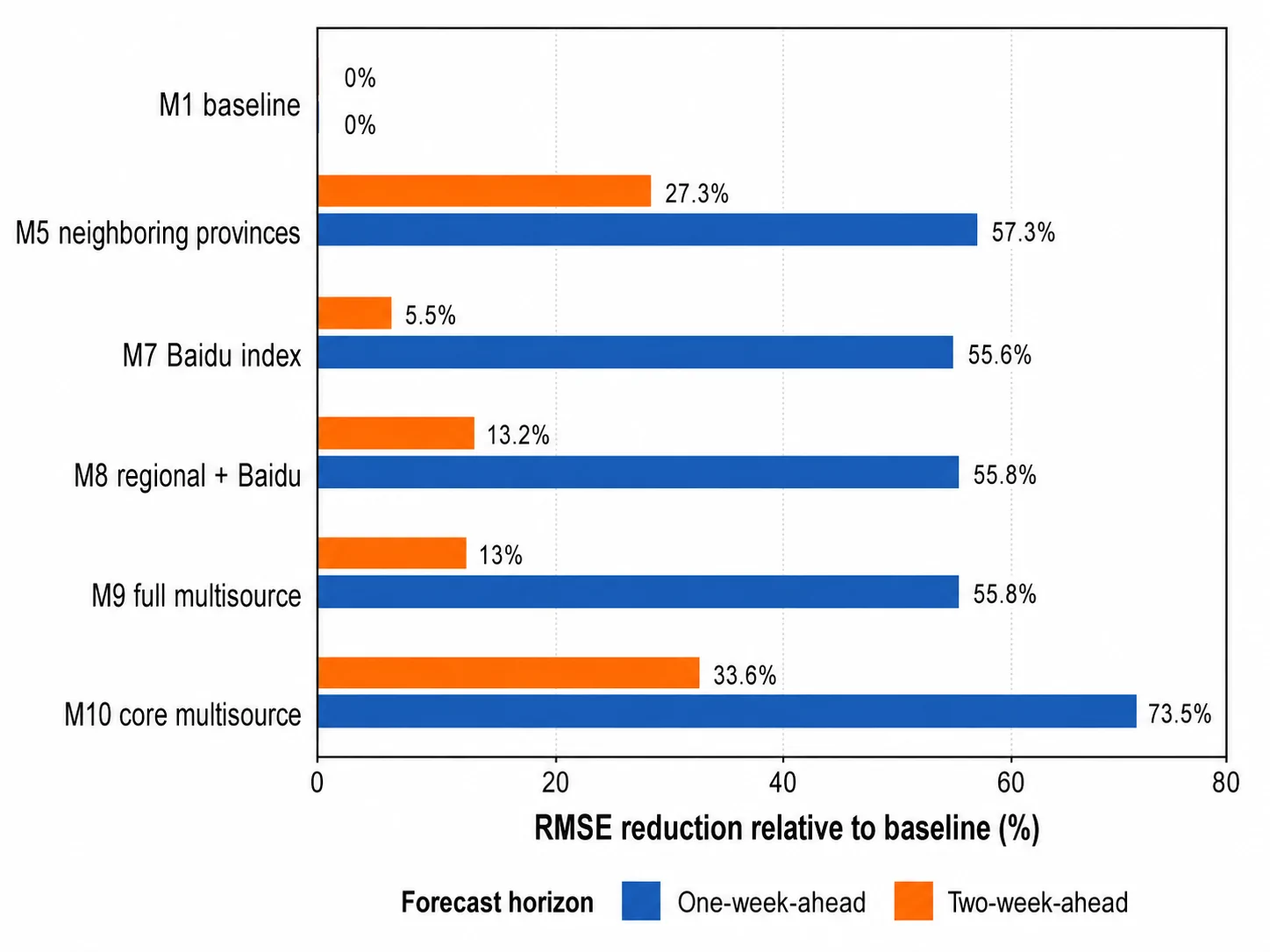
Robustness of model performance across 1-week-ahead and 2-week-ahead forecasts. RMSE: root mean squared error.

When the forecast horizon was extended to 2 weeks ahead, RMSE reductions decreased across models. The M10 core multisource model still achieved the largest RMSE reduction (33.6%), followed by the M5 neighboring-province pathogen model (27.3%). In contrast, the RMSE reductions of the M8 regional pathogen plus Baidu index model and the M9 full multisource model decreased to 13.2% and 13%, respectively, while the M7 Baidu index model showed only a 5.5% reduction. Overall, RMSE reductions were smaller for 2-week-ahead prediction than for 1-week-ahead prediction across all models. The M10 core multisource model showed the largest RMSE reduction in both the 1-week-ahead and 2-week-ahead prediction analyses, followed by the M5 neighboring province pathogen model in the 2-week-ahead analysis (Figure 7).

## Discussion

### Principal Findings

This study developed a multisource fusion early warning framework for respiratory infectious diseases in Beijing by integrating weekly reported case data, spatially stratified regional pathogen surveillance indicators, Baidu index search data, historical case trends, and seasonal terms. The main findings were as follows. First, weekly reported respiratory infectious disease cases in Beijing showed clear seasonal fluctuations, with a major winter peak and a secondary spring peak. Second, regional pathogen activity showed strong temporal associations with Beijing case counts. Overall pathogen positivity, coinfection rate, and coinfection-related positivity mainly showed same-week correlations, whereas influenza A positivity in several regional strata showed more evident leading correlation patterns. Third, in the 1-week-ahead prediction task, the M10 core multisource model achieved the lowest RMSE among all candidate models, indicating improved absolute case count prediction compared with the historical case baseline model. Fourth, the M7 Baidu index model achieved the lowest SMAPE, suggesting that search behavior indicators may capture relative temporal changes differently from models optimized for absolute case count prediction. Finally, when the forecast horizon was extended to 2 weeks ahead, predictive gains generally decreased across models, although M10 still maintained the largest RMSE reduction, followed by the M5 neighboring province pathogen model.

### Interpretation and Comparison With Previous Studies

The observed seasonal pattern was broadly consistent with previous evidence showing marked geographic heterogeneity in respiratory pathogen seasonality across China. Influenza activity in northern China is generally dominated by winter epidemics, whereas southern and intermediate-latitude regions may exhibit different peak timing or semiannual patterns [12,13]. Because the outcome in this study comprised multiple notifiable respiratory infectious diseases rather than influenza alone, the observed major winter peak and secondary spring peak may reflect the combined circulation of pathogens with different seasonal profiles. Previous work has shown that aggregate respiratory syndromic signals can represent the superposition of several pathogens whose timing and relative contributions vary across seasons [14]. This supports the inclusion of broad pathogen surveillance indicators when predicting aggregated respiratory infectious disease activity.

The temporal and lagged correlation results highlight the value of regional pathogen surveillance for understanding respiratory infectious disease activity in Beijing. Overall pathogen positivity and coinfection-related indicators showed their strongest correlations with Beijing case counts in the same week, suggesting that these indicators mainly reflected concurrent or near-concurrent regional epidemic intensity. In contrast, influenza A positivity showed more evident leading patterns in several regional strata, particularly in neighboring provinces and national non-Beijing regions. This finding is epidemiologically plausible because influenza epidemics exhibit spatial dependence, regional synchronization, and movement-driven transmission. Geographic distance, commuting patterns, long-distance population movement, and similarities in climate and seasonal conditions can influence the timing and pathways of epidemic spread [8,12,13,15]. Previous forecasting studies have also shown that models incorporating spatial and multiscale surveillance information can improve local or regional influenza forecasts compared with models relying solely on the historical observations of the target location [8,16,17]. These findings support the use of spatially stratified pathogen surveillance indicators rather than exclusive reliance on locally reported cases.

The model comparison further clarified the predictive value of pathogen information at different spatial scales. In the 1-week-ahead prediction task, M3, the national non-Beijing pathogen model, ranked second among all candidate models according to RMSE. This finding indicates that broad background pathogen activity outside Beijing contained useful information for predicting respiratory infectious disease activity in the city. Nationally aggregated indicators should therefore not be interpreted as uninformative or necessarily inferior to indicators constructed from geographically smaller areas. A recent assessment of spatial information in influenza forecasting similarly found that incorporating either neighboring state activity or a national population-weighted average produced modest improvements over models containing only lagged local activity, while neighboring information did not consistently outperform national background information [17]. Taken together, these results suggest that broad national signals may capture shared seasonal forcing, synchronized pathogen circulation, and epidemic conditions extending beyond immediately adjacent regions.

At the same time, M5, the neighboring province pathogen model, and M4, the northern region pathogen model, remained epidemiologically meaningful. These indicators may better represent geographic proximity, interregional mobility, transportation connectivity, climatic similarity, and winter-spring respiratory pathogen circulation relevant to Beijing [8,12,13,15]. The relatively strong performance of M6, the southern control model, also deserves careful interpretation. Although this model was originally designed as a geographically distant reference, its performance suggests that southern region indicators may capture broad national seasonal activity, shared pathogen trends, or epidemic phases that are correlated across regions. It should therefore not be interpreted as a strict negative control model. More generally, the results indicate that respiratory infectious disease prediction in megacities may benefit from both broad background signals and spatially structured regional signals. Their relative contributions may vary according to the forecast horizon, evaluation metric, pathogen composition, seasonal phase, and degree of national epidemic synchronization [13,16,17].

Baidu index indicators provided additional digital surveillance information, but their role should be interpreted cautiously. In the 1-week-ahead prediction task, M7, the Baidu index model, achieved the lowest SMAPE, suggesting that internet search behavior may be useful for capturing relative temporal changes, symptom-related information seeking, or short-term public attention associated with respiratory symptoms and disease names. Previous studies in China have identified temporal associations between Baidu search queries and influenza activity and have shown that combining search data with conventional influenza-like illness or virological surveillance data can improve short-term prediction under some settings [6,7,18-20]. These findings support the use of search behavior as a timely complementary signal.

However, M7 produced a smaller RMSE reduction than M10, and its predictive improvement decreased markedly in the 2-week-ahead sensitivity analysis. Internet search behavior may reflect immediate symptom perception, disease-related concern, health care–seeking interest, and public risk awareness, but it can also be affected by media coverage, policy changes, major public events, platform algorithms, and changes in the way people seek health information [1,6,10,11]. Moreover, the predictive value of search terms may differ across geographic regions and epidemic periods, requiring region-specific feature selection and repeated model updating [6,18-20]. Baidu index data should therefore be regarded as an auxiliary digital surveillance signal rather than a standalone replacement for laboratory-based pathogen surveillance or routinely reported case surveillance.

The M10 core multisource model achieved the lowest RMSE among all candidate models, but it did not achieve the lowest SMAPE. This discrepancy indicates that RMSE and SMAPE capture different dimensions of forecast performance. RMSE gives greater weight to large absolute errors and is therefore particularly relevant when the objective is to predict absolute case burden during high-activity periods, when inaccuracies may affect health care capacity planning, laboratory testing demand, and public health resource allocation. In contrast, SMAPE is a relative error measure whose behavior depends on the magnitudes of both the observed and predicted values and may differ substantially from RMSE during weeks with relatively low case counts. Previous methodological research has emphasized that commonly used forecast accuracy measures have different statistical properties and that reliance on a single metric may lead to incomplete or potentially misleading model comparisons [21]. The burden-stratified analysis further clarified this discrepancy. In high-burden test weeks, M10 substantially reduced RMSE and MAE compared with the M1 baseline model, indicating that its overall RMSE advantage was mainly driven by improved absolute case count prediction during periods of high disease activity. However, M10 did not perform best within every burden stratum, and its RMSE and SMAPE were higher than those of the baseline model in low-burden weeks. Therefore, the advantage of M10 should be interpreted primarily as improved absolute burden prediction during high-activity periods rather than uniform superiority across all weeks or all performance metrics. Future operational evaluations should additionally consider directional accuracy, peak timing error, peak intensity error, probabilistic calibration, prediction interval coverage, and threshold-based warning performance.

The predictors selected by M10 further support the importance of regional pathogen surveillance. Although Baidu index indicators were included in the candidate predictor set of the core multisource model, the final nonzero coefficients were dominated by regional pathogen indicators, including overall pathogen positivity, influenza A positivity, and coinfection-related indicators, together with a small number of historical case and seasonal terms. This suggests that, within the rolling-origin cross-validated LASSO-Poisson framework, the main predictive contribution of M10 was derived from spatially structured pathogen surveillance signals rather than unrestricted stacking of all available variables. Several selected predictors had negative coefficients, including some lagged or same-week pathogen positivity indicators. These negative coefficients should be interpreted cautiously and should not be viewed as evidence of causal protective effects. In the LASSO-Poisson model, coefficients represent conditional predictive contributions after adjustment for other selected lagged variables, historical case terms, and seasonal terms. Because many pathogen indicators were temporally correlated and shared similar winter-spring seasonal patterns, negative coefficients may reflect multicollinearity, seasonal adjustment, suppression effects, or LASSO shrinkage among correlated predictors. For example, a negative coefficient for same-week Beijing influenza A positivity may indicate an inverse residual association with next-week aggregated respiratory disease counts after adjustment for historical cases, regional influenza A activity, and other pathogen indicators; it should not be interpreted as evidence that influenza A activity reduces subsequent respiratory infections. Although pathogen interference or competitive dynamics between respiratory viruses are biologically plausible, the aggregated ecological design of this study cannot distinguish such mechanisms from statistical adjustment effects. Future pathogen-specific analyses with longer time series are needed to examine potential pathogen-pathogen interactions.

LASSO regularization performs coefficient shrinkage and variable selection by imposing an L1 penalty, thereby allowing some coefficients to be reduced exactly to zero [22]. Efficient regularization path algorithms extend this approach to generalized linear models, including penalized count regression settings [23]. The better RMSE performance of M10 compared with M9, the full multisource model, is consistent with the rationale for regularization: adding a larger number of candidate predictors, particularly highly correlated lagged variables, does not necessarily improve out-of-sample prediction. A parsimonious set of epidemiologically interpretable indicators may provide greater predictive stability and clearer operational interpretation than unrestricted inclusion of all available signals. Nevertheless, the selected LASSO coefficients represent conditional predictive contributions under a penalized modeling framework. They should not be interpreted as causal effects, and variable selection among correlated predictors may depend on the study period and penalty parameter.

The 2-week-ahead sensitivity analysis showed that the predictive gains associated with external surveillance indicators decreased as the forecast horizon increased. M10 retained the largest RMSE reduction at 2 weeks ahead, followed by M5, whereas the gains associated with the Baidu index model, the regional pathogen plus Baidu model, and the full multisource model decreased substantially. This pattern suggests that the predictive value of multisource surveillance indicators was strongest for near-term forecasting and weakened as the temporal distance between the available predictors and the target week increased. Recent studies combining Baidu index data with traditional influenza surveillance have similarly reported their best performance for shorter forecast horizons, followed by declining accuracy as the prediction horizon extends [18-20]. This may reflect the rapid decay of information contained in symptom-related searches and current pathogen activity, as well as the accumulation of uncertainty regarding future transmission, population behavior, and pathogen interactions.

The relatively stable performance of the neighboring province pathogen model in the 2-week-ahead prediction task further suggests that pathogen activity in geographically connected regions may provide a more persistent upstream signal than digital search behavior. However, this interpretation should remain cautious because predictive relationships may vary across epidemic phases and respiratory seasons. Forecasting studies have shown that the value of spatial information is heterogeneous both within and between seasons [17]. Additional multiseason and prospective validation is therefore required to determine whether neighboring province indicators consistently provide longer leading information or whether their observed value was specific to the epidemic patterns covered by the present dataset.

The findings also reinforce the value of monitoring multiple respiratory pathogens rather than modeling aggregated respiratory disease activity as if it represented a single transmission process. Previous research has shown that decomposing influenza-like illness into pathogen-specific components and then aggregating the corresponding forecasts can improve the prediction of both near-term and seasonal outcomes [14]. In addition, surveillance network studies have demonstrated that collective information from multiple locations can support accurate estimation and forecasting of respiratory virus activity, even in locations with limited direct observations [24]. In the present study, overall pathogen positivity, influenza A positivity, coinfection rate, and coinfection-related positivity captured different aspects of regional respiratory pathogen circulation. Their joint consideration may therefore provide a more comprehensive representation of epidemic intensity than any single pathogen indicator.

### Public Health Implications

From a public health perspective, this study supports the development of respiratory infectious disease early warning systems that integrate local case surveillance with regional pathogen surveillance and digital behavioral signals. For megacities such as Beijing, relying solely on locally reported cases may limit the ability to identify external transmission context and upstream changes in pathogen activity. Incorporating spatially stratified pathogen indicators may help surveillance systems identify periods of increasing case burden, adjust pathogen testing strategies, and improve preparedness for health care and laboratory resource demand. Baidu index data may further supplement information on symptom perception and disease-related public attention. More broadly, evidence from multisource forecasting research suggests that complementary information streams can improve surveillance timeliness, although not every additional source necessarily contributes independent predictive value [2,6,14,18,24]. In practice, the outputs of such models should be interpreted together with routine epidemiological surveillance, pathogen testing results, situational assessments, and expert judgment rather than being used as fully automated decision rules.

### Limitations

This study has several limitations. First, it was based on weekly aggregated data and used an ecological time series design. The results therefore represent population-level statistical associations and cannot be used to infer individual infection risks or causal relationships. Second, pathogen surveillance data may be influenced by testing strategies, laboratory capacity, the composition of tested populations, and differences in testing volume across regions and weeks. Although positivity rates were calculated as the total number of positive detections divided by the total number of specimens tested within each region-week, changes in testing behavior and population coverage could not be completely excluded. Third, Baidu index data may be affected by media coverage, public attention, policy changes, platform algorithms, and changes in internet information-seeking behavior, potentially limiting their stability as digital surveillance indicators [1,6,10,11]. Fourth, the study did not include other potentially relevant predictors, such as meteorological conditions, population mobility, outpatient or emergency department visits, vaccination coverage, school calendars, or prevention and control policies. Some apparent predictive information in the regional pathogen indicators may therefore represent shared seasonal or mobility-related factors that were not directly modeled. Fifth, a unified LASSO-Poisson framework was intentionally used to compare the incremental value of different surveillance data sources under a consistent modeling structure. The study did not systematically compare nonlinear machine learning, ensemble, mechanistic, or deep learning approaches, some of which may capture complex interactions and nonlinear temporal dependencies [6,16,18-20]. The objective of the present analysis was source comparison and interpretability rather than exhaustive algorithm comparison. Sixth, LASSO coefficients reflect predictive associations after penalized multivariable adjustment and should not be interpreted as causal effects [22,23]. Selection of individual variables may also be sensitive to correlations among lagged predictors and to temporal changes in epidemic dynamics. Finally, this study was conducted using data from Beijing and did not include external temporal or spatial validation. Beijing has distinct demographic characteristics, population mobility patterns, health care access, surveillance infrastructure, and reporting practices, which may influence both respiratory disease dynamics and the performance of multisource surveillance indicators. Therefore, the present findings provide initial evidence of feasibility and predictive value in Beijing, but prospective temporal validation and external validation in other urban settings are needed before broader application.

### Conclusions

In conclusion, spatially stratified regional pathogen surveillance indicators provided important supplementary information for 1-week-ahead prediction of respiratory infectious disease case counts in Beijing. The M10 core multisource model achieved the lowest RMSE, and its selected predictors were dominated by regional pathogen activity, particularly overall pathogen positivity, influenza A positivity, and coinfection-related indicators. National non-Beijing pathogen activity also demonstrated substantial predictive value, indicating that broad national background signals and geographically structured regional signals should be viewed as complementary rather than mutually exclusive. Baidu index data provided additional digital information, particularly for relative prediction performance, but should be interpreted as an auxiliary signal. These findings support the feasibility of an exploratory multisource prediction framework in Beijing that integrates local surveillance, regional pathogen activity, digital search behavior, historical case trends, seasonal structure, and expert interpretation to strengthen preparedness for urban respiratory infectious diseases. Further prospective temporal validation and spatial validation in other megacities are needed before broader application.

## Supplementary material

10.2196/100492 Multimedia Appendix 1 Definition of regional groups used to construct pathogen surveillance indicators.

10.2196/100492 Multimedia Appendix 2 Grouping of Baidu index search terms and their analytical roles.

10.2196/100492 Multimedia Appendix 3 Rolling-origin cross-validation settings and selected lambda values for candidate LASSO (least absolute shrinkage and selection operator)–Poisson models.

10.2196/100492 Multimedia Appendix 4 Burden-stratified prediction performance of candidate models in high-burden and low-burden weeks.

## Abbreviations

GISRS: Global Influenza Surveillance and Response System
LASSO: least absolute shrinkage and selection operator
MAE: mean absolute error
NREVSS: National Respiratory and Enteric Virus Surveillance System
RMSE: root mean squared error
SMAPE: symmetric mean absolute percentage error
